# Novel key genes in the pathogenesis of recurrent pelvic organ prolapse identified via bioinformatics analysis

**DOI:** 10.1097/MD.0000000000045134

**Published:** 2025-10-03

**Authors:** Wenhua Liu, Yue Zhang

**Affiliations:** a Department of Obstetrics and Gynecology, Hangzhou Women’s Hospital, Hangzhou, China.

**Keywords:** bioinformatics analysis, differentially expressed gene, recurrent pelvic organ prolapse

## Abstract

The causes of pelvic organ prolapse (POP) recurrence are sufficiently understood. Few studies have investigated the key genes of the recurrence of POP. In the present study, we screened the hub genes responsible for the recurrence of POP. The GSE28660 gene expression dataset contained microarray data of 4 recurrent POP and 4 primary POP uterosacral ligaments. We used the online gene expression omnibus microarray expression profiling dataset to identify differentially expressed genes. Further analyses of functional enrichment and protein-protein interaction networks (PPIs) were conducted, and key modules were identified. In the next step, we used the CIBERSORT algorithm to investigate differences in immune cell infiltration between recurrent and primary POP tissues. A total of 84 upregulated genes and 32 downregulated genes were identified. DNA microarray analysis of the human genome identified 116 genes associated with recurrence POP, and 2 hub genes, including cell death-inducing DFFA-like effector (CIDEA) and hemoglobin subunit delta (HBD), may contribute to the pathogenesis of recurrence POP, potentially providing diagnostic and therapeutic value.

## 1. Introduction

Pelvic organ prolapse (POP), a prevalent condition indicative of pelvic floor dysfunction, is estimated to affect 30% to 50% of women over the age of 50, with its prevalence projected to reach 46% by 2050.^[1]^ Despite advances in surgical treatment, recurrence rates remain notably high, imposing a substantial clinical and economic burden. A large retrospective cohort study spanning 2 decades and involving 1811 patients reported a reoperation rate of 5.1 per 1000 women-years, with a cumulative incidence of 5.6%.^[2]^ Known risk factors for POP include parity, vaginal delivery, advanced age, elevated body mass index, and advanced preoperative stage.^[3]^ Although previous research has implicated pathways such as those involving nerve growth factor in the initial development of POP,^[4]^ the molecular mechanisms driving its recurrence remain profoundly elusive. This gap in knowledge underscores the critical need to identify the specific genetic and cellular alterations that lead to treatment failure, which is essential for developing more effective preventive and therapeutic strategies.

The uterosacral ligaments (USLs) are critical structures for uterine support, and their functional integrity relies on the composition and strength of the extracellular matrix (ECM).^[5,6]^ In POP, these tissues exhibit significant declines in both the quality and quantity of collagen and elastic ECM proteins. Modern bioinformatics approaches have become indispensable for mining genomic data to uncover disease-specific signatures.^[7,8]^ In particular, Protein-Protein Interaction (PPI) network analysis can reveal functional modules and hub genes central to disease pathogenesis.^[9]^ Furthermore, algorithms such as CIBERSORT enable the deconvolution of immune cell infiltration from bulk transcriptome data, offering insights into the tumor microenvironment or, in this context, the stromal immune landscape.^[10]^

While recent bioinformatic studies, such as the analysis by Chen et al have explored gene expression changes involved in the pathogenesis of primary POP,^[11]^ the distinct molecular profile of recurrent POP remains uncharted territory. Therefore, the present study aims to address this specific gap by performing a comprehensive bioinformatics analysis on the GSE28660 dataset, which uniquely includes samples from patients with recurrent POP. By integrating differential gene expression analysis, PPI network construction, and immune infiltration assessment, we seek to identify hub genes and pathways specifically associated with POP recurrence, thereby offering novel diagnostic and therapeutic perspectives.

## 2. Methods

### 2.1. Microarray data

The microarray expression profiling dataset GSE28660, deposited by Eyster et al., was downloaded from the Gene Expression Omnibus (GEO, https://www.ncbi.nlm.nih.gov/ A new algorithm for enumeration of immune cell subset,^[11]^ CIBERSORT (https://cibersort.stanford.edu/), provides the possibility to identify immune biomarkers for diagnosis and prognosis.^[12]^ Our study found hub genes and immune cells highly related to recurrent POP by analyzing expression spectrum data in public databases using (PPI) network analysis and the CIBERSORT algorithm, providing novel ideas and methods for the treatment of recurrence POP.geo/). The dataset was based on the GPL2895 GE Healthcare/Amersham Biosciences CodeLink Human Whole Genome Bioarray platform. The GSE28660 dataset, containing 4 primary POP and 4 recurrent POP patients, was the only dataset to meet this condition. GEO belongs to public databases. The patients involved in the database have obtained ethical approval. Users can download relevant data for free for research and publish relevant articles. Our study is based on open-source data, so there are no ethical issues or other conflicts of interest.

### 2.2. Differential expression analysis

Differential expression analysis was performed using the online analysis tool GEO2R; the expression profiles of primary POP and recurrent POP patients were compared to identify the differentially expressed genes (DEGs). *P*-values and adjusted *P*-values were calculated using *t*-tests. Significant DEGs were selected using an adjusted *P*-value of <.05 and |log2 fold change|>1. We selected the most significant genes when the DEGs were duplicated. The heatmap and Volcano map for the DEGs was created using were drawn using the hiplot tool (https://hiplot.com.cn).

### 2.3. Functional enrichment analysis of DEGs

The functional enrichment analysis of DEGs was performed with the DAVID Bioinformatics Tool (version 6.8, https://david-d.ncifcrf.gov/). This analysis included the functional categories, Gene Ontology (GO) terms, and Kyoto Encyclopedia of Genes and Genomes (KEGG) pathways. The GO analysis included 3 categories, namely, biological process, cellular component, and molecular function, which were used to predict protein functions. KEGG pathway analysis was used to assign sets of DEGs to specific pathways to enable the construction of molecular interaction, reaction, and relationship networks. Benjamini-adjusted *P* <.05 and an enriched gene count >5 were chosen as the criteria for significance.

### 2.4. PPI network analysis

The PPI network analysis was conducted using STRING (https://string-db.org/), which is an online database of known and predicted PPIs. These interactions include physical and functional associations, and the data are mainly derived from computational predictions, high-throughput experiments, automated text mining, and co-expression networks. We mapped the DEGs onto the PPI network and set an interaction score of >0.4 as the threshold value. In addition, Cytoscape v3.6.0 software was used to visualize and construct the PPI network. Nodes with the greatest number of interactions with neighboring nodes were considered hub nodes. To identify the key PPI network modules, the app Mcode from the Cytoscape software suite was used to perform the gene network clustering analysis. *P* <.05 was set as the significance threshold for identifying key modules.

### 2.5. Immune cell infiltration in POP tissues

The CIBERSORT algorithm was employed to elucidate the proportion of 22 immune cells in POP tissues. The samples with p-values <.05 were significant.^[8]^ Pearson correlation analysis was implemented to obtain the related coefficient between the 22 immune cells. Then, we investigated the differential immune cell infiltration between primary POP and recurrent POP tissues.

## 3. Results

### 3.1. Differentially expressed genes

**Figure 1.**
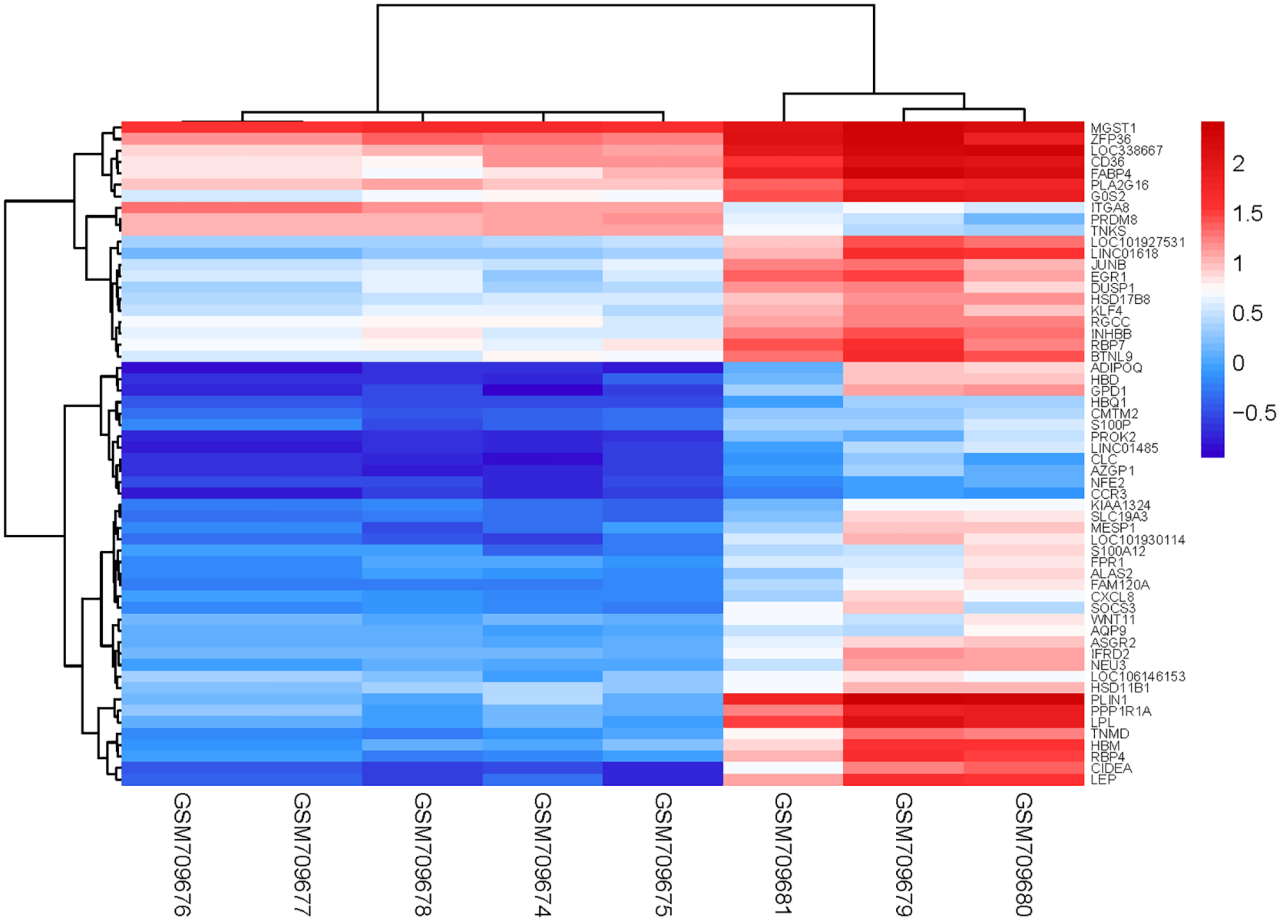
Heatmap of DEG clustering. Blue represents downregulation and red represents upregulation. DEG = diferentially expressed gene.

**Figure 2.**
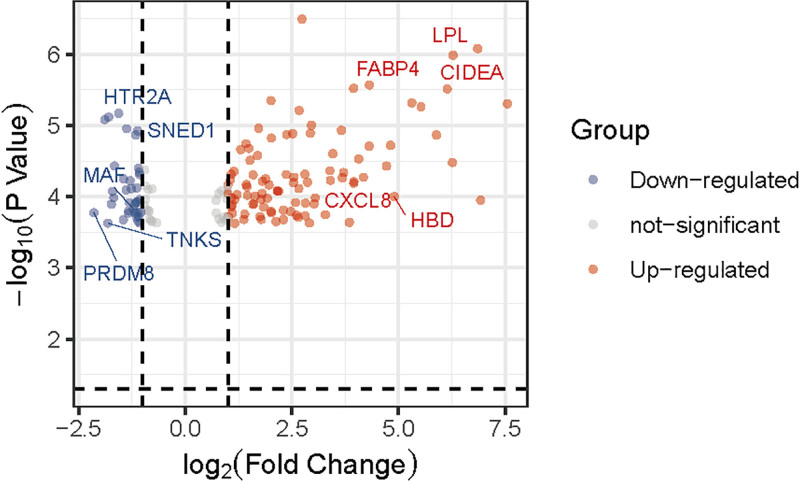
Volcano map of DEG clustering. Blue represents downregulation and red represents upregulation. DEG = diferentially expressed gene.

We downloaded the microarray expression dataset GSE28660 from the GEO database and analyzed the DEGs between primary POP and recurrent POP patients using the online analysis tool GEO2R. In total, 84 upregulated and 32 downregulated genes were identified in the differential expression analysis. The results were shown in Table 1. The heatmap and volcano map showed the differentially expression expressed genes in primary POP and recurrent POP groups. The top 20 genes-PLIN1, PPP1R1A, LPL, TNMD, HBM, RBP4, CIDEA, LEP, LOC101927531, LINC01618, JUNB, EGR1, DUSP1, HSD17B8, KLF4, RGCC, INHBB, RBP7, BTNL9, HBD were analyzed in a heatmap. The results were shown in Figures 1 and 2.

**Table 1:** Differentially expressed genes.

Gene symbol	Adjusted P-value	logFC	Gene title
Up regulated genes			
LEP	0.0228	7.554	Leptin
GPD1	0.0387	6.924	Glycerol-3-phosphate dehydrogenase 1
PLIN1	0.0174	6.855	Perilipin 1
LPL	0.0174	6.277	Lipoprotein lipase
ADIPOQ	0.0299	6.263	Adiponectin, C1Q and collagen domain containing
CIDEA	0.0221	6.141	Cell death-inducing DFFA-like effector a
RBP4	0.0228	5.527	Retinol binding protein 4
PPP1R1A	0.0228	5.312	Protein phosphatase 1 regulatory inhibitor subunit 1A
HBD	0.038	4.899	Hemoglobin subunit delta
LOC101930114	0.0256	4.815	Uncharacterized LOC101930114
TNMD	0.0319	4.718	Tenomodulin
FABP4	0.0221	4.316	Fatty acid binding protein 4
LINC01485	0.0256	4.311	Long intergenic nonprotein coding RNA 1485
HBM	0.0354	4.176	Hemoglobin subunit mu
AZGP1	0.0354	3.949	Alpha-2-glycoprotein 1, zinc-binding
PROK2	0.0221	3.948	Prokineticin 2
MESP1	0.0493	3.846	Mesoderm posterior bHLH transcription factor 1
LINC01618	0.0346	3.713	Long intergenic nonprotein coding RNA 1618
SLC19A3	0.0354	3.683	Solute carrier family 19 member 3
G0S2	0.0238	3.66	G0/G1 switch 2
LOC338667	0.0279	3.459	V-set and immunoglobulin domain-containing protein 10-like
SOCS3	0.0354	3.404	Suppressor of cytokine signaling 3
CLC	0.0342	3.388	Charcot-Leyden crystal galectin
CD36	0.0453	3.285	CD36 molecule
CXCL8	0.0384	3.048	C-X-C motif chemokine ligand 8
S100A12	0.0403	3.023	S100 calcium binding protein A12
BTNL9	0.0238	2.958	Butyrophilin like 9
S100P	0.0238	2.931	S100 calcium binding protein P
NEU3	0.0493	2.91	Neuraminidase 3
EGR1	0.0354	2.867	Early growth response 1
FAM120A	0.04	2.818	Family with sequence similarity 120A
KIAA1324	0.0462	2.812	KIAA1324
ZFP36	0.0346	2.805	ZFP36 ring finger protein
CMTM2	0.0162	2.737	CKLF like MARVEL transmembrane domain-containing 2
LOC101927531	0.0354	2.662	Uncharacterized LOC101927531
HBQ1	0.0465	2.645	Hemoglobin subunit theta 1
IFRD2	0.046	2.61	Interferon related developmental regulator 2
ALAS2	0.0399	2.588	5’-aminolevulinate synthase 2
TPO	0.0354	2.548	Thyroid peroxidase
RBP7	0.0354	2.539	Retinol binding protein 7
FPR1	0.0238	2.522	Formyl peptide receptor 1
DUSP1	0.044	2.474	Dual specificity phosphatase 1
JUNB	0.0238	2.377	JunB proto-oncogene, AP-1 transcription factor subunit
INHBB	0.0346	2.371	Inhibin beta B subunit
ASGR2	0.044	2.301	Asialoglycoprotein receptor 2
CCR3	0.0476	2.3	C-C motif chemokine receptor 3
HSD11B1	0.0354	2.161	Hydroxysteroid 11-beta dehydrogenase 1
NFE2	0.0354	2.156	Nuclear factor, erythroid 2
PLA2G16	0.0492	2.129	Phospholipase A2 group XVI
PADI4	0.0254	2.019	Peptidyl arginine deiminase 4
RGCC	0.044	2.018	Regulator of cell cycle
LOC106146153	0.044	2.01	Uncharacterized lncRNA LOC106146153
LOC101928284	0.0228	2.009	Uncharacterized LOC101928284
MGST1	0.0384	1.922	Microsomal glutathione S-transferase 1
AQP9	0.0354	1.809	Aquaporin 9
HSD17B8	0.0369	1.799	Hydroxysteroid 17-beta dehydrogenase 8
LINC01272	0.0476	1.781	Long intergenic nonprotein coding RNA 1272
WNT11	0.0384	1.781	Wnt family member 11
MIR193BHG	0.0462	1.753	MIR193B host gene
CSF3R	0.0238	1.72	Colony stimulating factor 3 receptor
KLF4	0.0279	1.693	Kruppel like factor 4
AHSP	0.038	1.637	Alpha hemoglobin stabilizing protein
MBP	0.0403	1.609	Myelin basic protein
OSM	0.0346	1.59	Oncostatin M
NR4A1	0.0403	1.585	Nuclear receptor subfamily 4 group A member 1
RAC3	0.0378	1.457	Ras-related C3 botulinum toxin substrate 3
SPTBN1	0.0354	1.431	Spectrin beta, non-erythrocytic 1
VNN2	0.0256	1.426	Vanin 2
ZZEF1	0.0447	1.395	Zinc finger ZZ-type and EF-hand domain-containing 1
STEAP1	0.0476	1.372	Six transmembrane epithelial antigen of the prostate 1
MRO	0.0262	1.3	Maestro
FUT7	0.0377	1.213	Fucosyltransferase 7
HADH	0.0384	1.157	Hydroxyacyl-CoA dehydrogenase
AMOTL2	0.0493	1.156	Angiomotin like 2
TSHZ2	0.0344	1.141	Teashirt zinc finger homeobox 2
LOC105376997	0.0407	1.104	Uncharacterized LOC105376997
GBA2	0.044	1.101	Glucosylceramidase beta 2
DNAJC3	0.0384	1.087	DnaJ heat shock protein family (Hsp40) member C3
MMP25	0.0346	1.078	Matrix metallopeptidase 25
MT1L	0.0417	1.077	Metallothionein 1L (gene/pseudogene)
TCF15	0.0354	1.058	Transcription factor 15 (basic helix-loop-helix)
RASL11A	0.0372	1.002	RAS like family 11 member A
RGS16	0.0354	1.001	Regulator of G-protein signaling 16
Down regulated genes			
ZNF740	0.044	−1.045	Zinc finger protein 740
AK5	0.0493	−1.062	Adenylate kinase 5
U2SURP	0.0384	−1.065	U2 snRNP associated SURP domain containing
RPS6KA5	0.0346	−1.073	Ribosomal protein S6 kinase A5
KRT18P59	0.0346	−1.079	Keratin 18 pseudogene 59
GUCY1B3	0.0462	−1.088	Guanylate cyclase 1 soluble subunit beta
SLF2	0.0332	−1.095	SMC5-SMC6 complex localization factor 2
FUS	0.0492	−1.109	FUS RNA binding protein
SNED1	0.0238	−1.115	Sushi, nidogen and EGF like domains 1
C8orf44	0.0399	−1.117	Chromosome 8 open reading frame 44
IDI1	0.044	−1.117	Isopentenyl-diphosphate delta isomerase 1
ERP29	0.0354	−1.134	Endoplasmic reticulum protein 29
NBEA	0.0465	−1.142	Neurobeachin
HLF	0.0399	−1.149	HLF, PAR bZIP transcription factor
LOC644656	0.0238	−1.162	Uncharacterized LOC644656
NAB2	0.0403	−1.175	NGFI-A binding protein 2
CATSPER2	0.044	−1.177	Cation channel sperm associated 2
MAF	0.0434	−1.216	MAF bZIP transcription factor
LOC399900	0.0354	−1.264	Uncharacterized LOC399900
C2orf50	0.0354	−1.293	Chromosome 2 open reading frame 50
TMEM35A	0.044	−1.331	Transmembrane protein 35A
CACNB3	0.0238	−1.373	Calcium voltage-gated channel auxiliary subunit beta 3
TBL1X	0.0354	−1.388	Transducin (beta)-like 1X-linked
DPY19L2P2	0.0354	−1.449	DPY19L2 pseudogene 2
CPA3	0.0476	−1.451	Carboxypeptidase A3
HTR2A	0.0228	−1.561	5-hydroxytryptamine receptor 2A
SGIP1	0.0319	−1.658	SH3 domain GRB2 like endophilin interacting protein 1
TMEM252	0.0354	−1.706	Transmembrane protein 252
ZNF680	0.0403	−1.734	Zinc finger protein 680
TNKS	0.0493	−1.814	Tankyrase
ITGA8	0.0233	−1.888	Integrin subunit alpha 8
PRDM8	0.044	−2.144	PR/SET domain 8

### 3.2. Functional and pathway enrichment of DEGs

**Figure 3.**
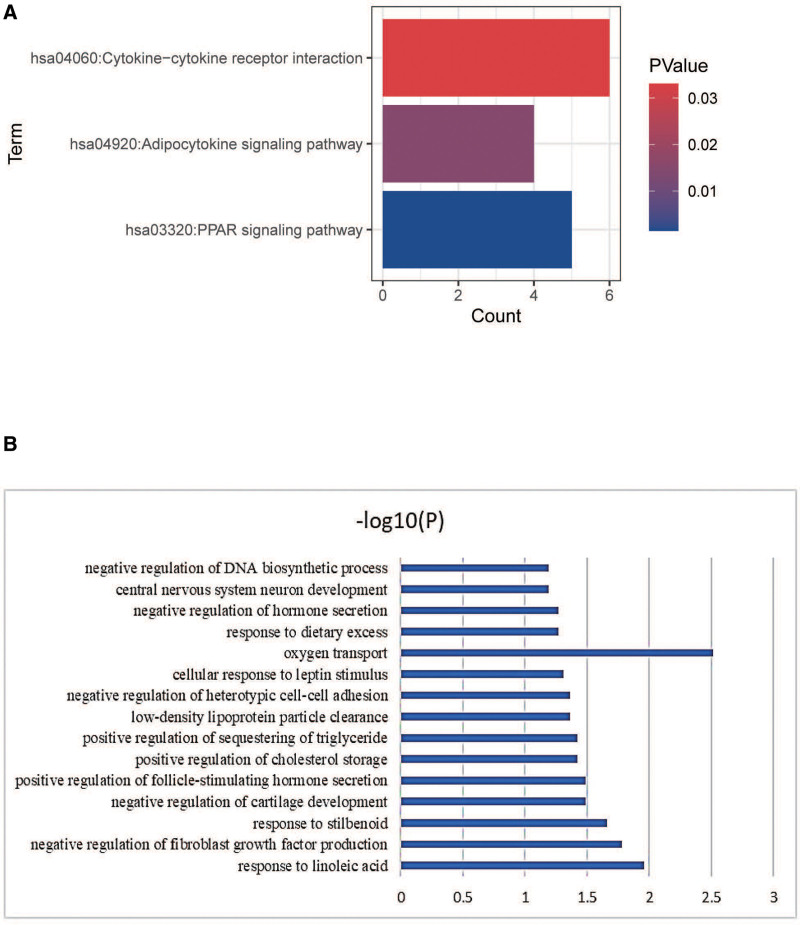
(A) Functional and pathway enrichment of DEG. (B) Bar graph of 15 representative enriched functional terms. The x-axis depicts the–log10 (*P*-value). The y-axis lists the enriched functional terms. DEG = diferentially expressed gene.

In our study, 17 enriched functional category terms, 30 enriched GO terms, and 3 KEGG pathways were identified. The enriched GO terms with *P* <.05 are presented in Table 2; they included positive regulation of transcription from RNA (*P* = .01951), negative regulation of transcription from RNA polymerase II promoter (*P* = .046058), intracellular signal transduction (*P* = .024445), inflammatory response (*P* = .018727), lipid metabolic process (*P* = .011286), chemotaxis (*P* = .004709)in the cellular component category. In addition, 6 enriched UP_KEYWORDS terms and 3 enriched UP_SEQ_FEATURE terms with *P* <.05 were identified, including Heme (*P* = 5.33E-04), NAD (*P* = 1.16E-02), Disulfide bond (*P* = 1.16E-02), Iron (*P* = 2.35E-02), Calcium (*P* = 3.17E-02), DNA-binding region: Basic motif (*P* = 1.41E-03), Disulfide bond (*P* = 1.74E-02), and Binding site: Substrate (*P* = 1.81E-02). The number of genes and P-values of the 15 enriched functional terms are displayed in Figure 3.

**Table 2:** The enriched terms for DEGs.

Category	Term	Count	Genes	*P*-value
UP_KEYWORDS	Heme	6	GUCY1B3, HBM, TPO, HBD, HBQ1, STEAP1	5.33 × 10^−04^
UP_SEQ_FEATURE	DNA-binding region:basic motif	6	MESP1, NFE2, HLF, MAF, TCF15, JUNB	1.41 × 10^−03^
UP_KEYWORDS	Lipid metabolism	9	HSD11B1, NEU3, PLA2G16, IDI1, LPL, PLIN1, HADH, HSD17B8, GBA2	1.50 × 10^−03^
UP_KEYWORDS	NAD	5	TNKS, GPD1, STEAP1, HADH, HSD17B8	1.16 × 10^−02^
UP_KEYWORDS	Disulfide bond	27	SNED1, CSF3R, CXCL8, FPR1, LPL, HTR2A, ASGR2, MMP25, TPO, FUT7, WNT11, KIAA1324, PROK2, CD36, CCR3, CPA3, BTNL9, PDIA2, ADIPOQ, OSM, INHBB, DNAJC3, AZGP1, RBP4, TNMD, LEP, ITGA8	1.73 × 10^−02^
UP_SEQ_FEATURE	Disulfide bond	24	BTNL9, CPA3, SNED1, PDIA2, CSF3R, CXCL8, ADIPOQ, OSM, FPR1, LPL, INHBB, HTR2A, ASGR2, MMP25, TPO, AZGP1, RBP4, FUT7, LEP, KIAA1324, ITGA8, PROK2, CD36, CCR3	1.74 × 10^−02^
UP_SEQ_FEATURE	Binding site:substrate	6	HSD11B1, NEU3, IDI1, GPD1, PADI4, HSD17B8	1.81 × 10^−02^
UP_KEYWORDS	Iron	6	GUCY1B3, HBM, TPO, HBD, HBQ1, STEAP1	2.35 × 10^−02^
UP_KEYWORDS	Calcium	10	SNED1, CACNB3, MMP25, TPO, CATSPER2, ITGA8, S100A12, S100P, PADI4, ASGR2	3.17 × 10^−02^
GOTERM_BP_DIRECT	Positive regulation of transcription from RNA polymerase II promoter	12	EGR1, MESP1, NR4A1, RPS6KA5, RGCC, HLF, MAF, TNKS, OSM, TBL1X, KLF4, JUNB	.01951
GOTERM_BP_DIRECT	Negative regulation of transcription from RNA polymerase II promoter	9	EGR1, ZFP36, MAF, LEP, TSHZ2, CD36, TBL1X, KLF4, JUNB	0.046058
GOTERM_BP_DIRECT	Intracellular signal transduction	7	GUCY1B3, ZFP36, RPS6KA5, CXCL8, PPP1R1A, DUSP1, RAC3	.024445
GOTERM_BP_DIRECT	Inflammatory response	7	MMP25, RPS6KA5, CXCL8, FPR1, PROK2, S100A12, CCR3	.018727
GOTERM_BP_DIRECT	Lipid metabolic process	5	LEP, CIDEA, LPL, PLIN1, CD36	.011286
GOTERM_BP_DIRECT	Chemotaxis	5	CXCL8, FPR1, PROK2, CMTM2, CCR3	.004709

DEGs = differentially expressed genes.

### 3.3. PPI network analysis of DEGs

**Figure 4.**
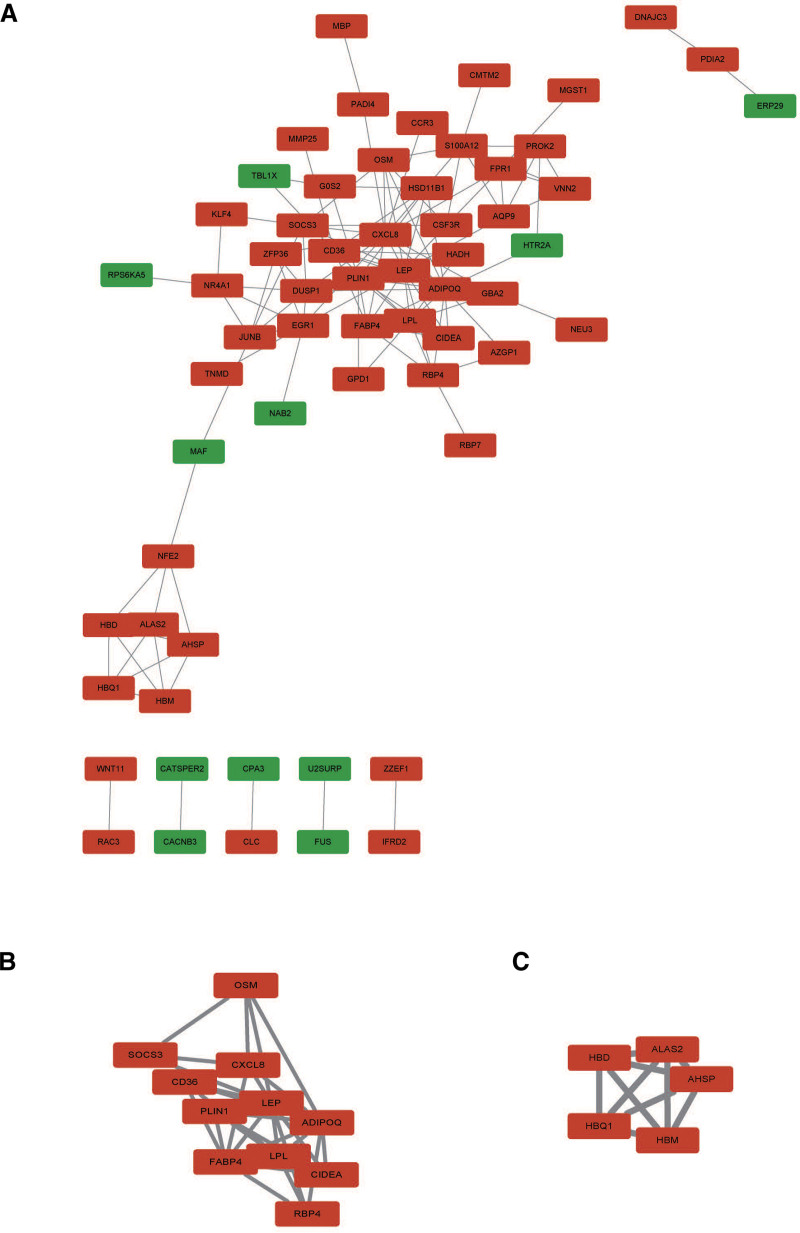
(A) PPI network visualized using Cytoscape software (version 3.6.0, https://cytoscape.org) based on interactions retrieved from the STRING database (version 11.5, https://string-db.org; interaction score >0.4). Cytoscape network visualization of the 34 nodes and 53 edges that were obtained with interaction scores >0.4 according to the STRING online database. The nodes represent genes, and the edges represent links between genes. Red represents upregulated genes, and green represents downregulatedgenes. (B and C) (Key Modules): Key modules identified within the PPI network using the MCODE plugin for Cytoscape. Two key modules were identified by Mcode, which was used to identify network gene clustering. PPI = protein–protein interaction.

A PPI network with 34 nodes and 53 edges was obtained; the network had an interaction score >0.4 according to the STRING online database (Fig. 4A). The nodes correspond to genes, and the edges represent the links between genes. Red represents upregulated genes, and green represents downregulated genes. We used Mcode in Cytoscape to perform network gene clustering to identify the key PPI network modules. As shown in Figure 4B and C, 2 key modules with 2 upregulated genes (CIDEA and HBD) were identified. Furthermore, functional enrichment analysis indicated that these 2 genes were mainly involved in activating apoptosis, immune response, and inflammation.

### 3.4. Identification of the key genes

We used Mcode in Cytoscape to perform network gene clustering to identify the key PPI network modules. As shown in Figure 4B and C, 2 key modules with 2 upregulated genes (CIDEA and HBD) were identified. Furthermore, functional enrichment analysis indicated that these 2 genes were mainly involved in activating apoptosis, and oxygen transport.

### 3.5. Immune cell infiltration analysis

**Figure 5.**
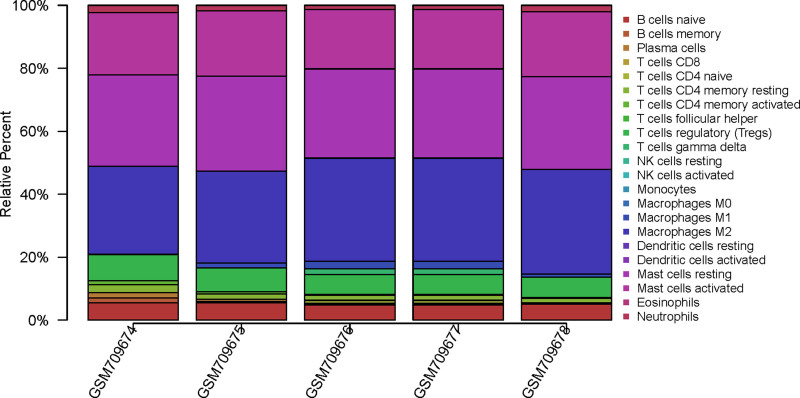
The landscape of immune cell infiltration in GSE28660 (CIBERSORT *P*-value <.05). Proportion of the 22 immune cell types in GSE28660.

The CIBERSORT algorithm was employed to select samples with an output *P* <.05. A total of 5 samples including primary POP and recurrent POP tissues were obtained. A bar plot was generated to show the proportion of 22 immune cells in the 5 samples. We found that there was no significant difference between the 22 immune cells in the 5 samples (Fig. 5).

## 4. Discussion

Recent advancements in bioinformatics have provided powerful tools for elucidating the molecular underpinnings of complex diseases. In this study, we analyzed the GSE28660 dataset to identify key genes and pathways differentially expressed in recurrent POP compared to primary POP. We identified 116 DEGs, with CIDEA and HBD emerging as the most significant hub genes through PPI network analysis. Functional enrichment analyses suggested that these genes and the broader set of DEGs are involved in biological processes such as apoptosis, oxygen transport, and inflammatory response, which may contribute to the weakening of pelvic support structures.

Our findings both align and contrast with recent literature. A previous bioinformatic study by Chen et al investigated biological changes in POP, providing valuable insights into the pathogenesis of primary POP.^[11]^ However, our study specifically focuses on the elusive mechanism of recurrence, a distinct clinical challenge. The identification of CIDEA and HBD as hub genes for recurrence is a novel finding. CIDEA, a gene known to interfere with energy metabolism and induce apoptosis,^[13–15]^ could potentially contribute to the progressive deterioration of pelvic floor muscle and ligament integrity. Similarly, the upregulation of HBD, often associated with inflammatory responses,^[16]^ aligns with the growing evidence that immunoregulatory processes play a crucial role in ECM remodeling following pelvic floor injury.^[17,18]^ This suggests that recurrent POP may be driven by a persistent cycle of apoptosis and inflammation, impairing proper tissue repair.

Given the close relationship between hub genes and immune processes, we investigated differences in the immune microenvironment using the CIBERSORT algorithm. Interestingly, no significant differences in immune cell infiltration were found between primary and recurrent POP tissues in our analysis. This negative result may be attributed to the limited sample size, which is a major limitation of this study. Larger-scale studies are necessary to conclusively determine the role of immune cells in recurrence. This notion is supported by other research that has identified immune-related hubs in primary POP^[16]^ and demonstrated the involvement of both immune and nonimmune cells in ECM dysregulation.^[19]^

Our study has several limitations. First, the analysis relied solely on a public microarray dataset with a very small sample size (n = 8), which limits the statistical power and generalizability of our findings. Second, as a bioinformatics investigation, our conclusions remain hypothetical and require validation through experimental models such as qPCR, western blot, or immunohistochemistry on clinical samples from recurrent POP patients. Future studies with larger, independent cohorts and functional experiments are essential to confirm the roles of CIDEA and HBD in the pathogenesis of recurrent POP.

## 5. Conclusion

In summary, this bioinformatics study identified 116 DEGs and pinpointed CIDEA and HBD as potential key hub genes in the pathogenesis of recurrent POP. Functional enrichment analyses suggest that processes such as apoptosis, inflammation, and oxygen transport may be involved in POP recurrence. The PPAR signaling pathway, adipocytokine signaling pathway, and cytokine-cytokine receptor interaction were also implicated. These findings provide novel, hypothesis-generating insights into the molecular mechanisms underlying POP recurrence. However, it is crucial to emphasize that these results are derived from in silico analysis of a small dataset. Therefore, the proposed genes and pathways represent promising candidates for future investigation rather than established mechanisms. Further validation with larger clinical cohorts and functional experiments is imperative to confirm their roles. Ultimately, this study lays a groundwork for future research aimed at developing targeted strategies for predicting and preventing the recurrence of POP.

## Acknowledgments

We thank the researchers who generated and uploaded the GSE28660 dataset to the public repository.

## Author contributions

**Conceptualization:** Wenhua Liu.

**Data curation:** Wenhua Liu.

**Formal analysis:** Wenhua Liu.

**Funding acquisition:** Wenhua Liu.

**Investigation:** Wenhua Liu

**Methodology:** Wenhua Liu.

**Project administration:** Wenhua Liu.

**Resources:** Wenhua Liu.

**Software:** Wenhua Liu.

**Supervision:** Wenhua Liu.

**Validation:** Wenhua Liu.

**Visualization:** Wenhua Liu.

**Writing – original draft:** Wenhua Liu.

**Writing – review & editing:** Yue Zhang.
